# Systemic Lupus Erythematosus Flare Masquerading as Bilateral Lower Extremity Non-pitting Edema: A Case Report

**DOI:** 10.7759/cureus.48344

**Published:** 2023-11-06

**Authors:** Ayesha Khan, Hala Mouhydeen, Hussein Gharib

**Affiliations:** 1 School of Medicine, St. George's University, St. George, GRD; 2 Internal Medicine, Ascension St. John Hospital, Detroit, USA

**Keywords:** multisystem sle, non-pitting edema, systemic lupus erythematosus, auto immune rheumatological conditions, sle flare

## Abstract

Systemic lupus erythematosus (SLE) is a complex autoimmune disease that affects multiple organ systems in the body. We report the case of a patient with new-onset bilateral lower extremity (LE) non-pitting edema as the only presenting symptom of a severe SLE flare. Other potential etiologies of non-pitting LE edema in patients with SLE were excluded, including hypothyroidism and lymphedema. Laboratory investigations and SLE disease activity index (SLEDAI) score suggested severe SLE flare. The edema improved with steroids and diuresis. Clinicians should know that non-pitting LE edema can be the only manifestation of a multisystem SLE flare.

## Introduction

Systemic lupus erythematosus (SLE) is a chronic autoimmune inflammatory disease that affects multiple organ systems. While the disease is more prevalent in women of childbearing age, African-American and Hispanic women are at the highest risk [1]. The initial presentation of SLE varies from mild photosensitive dermatologic rashes and musculoskeletal pain to life-threatening involvement of the central nervous system, renal, cardiovascular, and pulmonary systems [2]. Early diagnosis and appropriate treatment are crucial to minimize long-term damage to organs and improve patient outcomes.

## Case presentation

**Figure 1:**
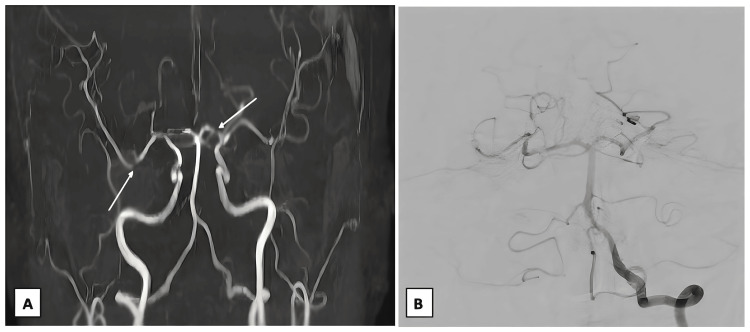
(A) Magnetic resonance angiogram of the circle of Willis conducted six months prior to the current presentation, showing cerebral vasculitis involving the cerebral arteries bilaterally. (B) Diagnostic cerebral angiogram showing no vasculitic changes during the patient's current hospitalization.

A young adult African-American female presented to the emergency department (ED) with complaints of bilateral lower extremity (LE) edema for one week. Her past medical history is significant for recently diagnosed SLE with neuropsychiatric manifestation of cerebral vasculitis (Figure 1), small bilateral subdural hematomas six months ago, as well as two spontaneous abortions. She was placed on hydroxychloroquine but was non-compliant. During her current presentation, a review of systems was negative for neurological, respiratory, musculoskeletal, or cardiac symptoms. On arrival, the patient was afebrile and saturating at 100% on room air. Her blood pressure was elevated at 147/100 mmHg, her heart rate was 75 beats per minute, and her respiratory rate was 15 breaths per minute. Physical examination revealed alopecia, discoid lesions of the chest, and bilateral LE non-pitting edema extending to the knees. There was no evidence of joint swelling or erythema. An S3 heart sound was heard upon auscultation. 

EKG showed sinus tachycardia with a rate of 128 along with new T wave inversions in leads I, II, avL, and V4-V6 when compared to an EKG six months ago. Lab work showed leukopenia, anemia, hypocalcemia, and a mildly low bicarbonate level. Urinalysis showed proteinuria as well as hematuria (Table 1). Chest X-ray showed cardiomegaly and left lower lobe linear atelectasis. A duplex ultrasound of the LE was negative for deep vein thrombosis (DVT) bilaterally. D-dimer was elevated at 3560. CT angiography (CTA) was positive for bilateral pulmonary emboli, moderate-sized pericardial effusion, and bilateral axillary lymphadenopathy. The patient’s LE edema, proteinuria, bicytopenia, pulmonary emboli, and pericardial effusions raised concern for a severe SLE flare with potential progression to lupus nephritis and serositis. Given the acuity of these findings, the patient was hospitalized for further evaluation and management. 

**Table 1:** Lab workup on presentation and upon discharge BUN, blood urea nitrogen; ESR, erythrocyte sedimentation rate; CRP, C-reactive protein; BNP, brain natriuretic peptide; HPF, high-power field; AST, aspartate aminotransferase; ALT, alanine aminotransferase; IU, international units; H, high; L, low; N/A, not available.

Lab workup	Normal range	On presentation	Upon discharge
WBC (k/mcL)	4.5-11	3.07 (L)	4.07
RBC (million/mcL)	4.35-5.65	3.39 (L)	3.11 (L)
Platelet (k/mcL)	150-400	167	254
BUN (mg/dL)	7-20	17	40
Creatinine (mg/dL)	0.7-1.3	0.97	2.04 (H)
ESR (mm/h)	0-15	61 (H)	N/A
CRP (mg/L)	<10	9.5	N/A
Pro-BNP (pg/mL)	<125	5,041 (H)	N/A
Ferritin (ng/mL)	12-150	613 (H)	3,090 (H)
Urine microalbumin (mg/dL)	<2	261.9 (H)	N/A
Urine random protein (mg/dL)	0-20	509 (H)	N/A
Urine RBC (RBC/HPF)	<4	26 (H)	N/A
AST (IU/L)	0-35	24	60 (H)
ALT (units/L)	0-40	11	13

The patient’s SLE disease activity index (SLEDAI) score was elevated at 15, suggesting severe SLE flare. A rheumatologic workup was pursued and showed positive antinuclear antibodies (ANA) and anti-dsDNA antibodies along with low C3 and C4 complement levels (Table 2). An echocardiogram revealed left ventricular diastolic impairment, with an ejection fraction of 15-20%. Additionally, there was severe global hypokinesis and a moderate-to-large, free-flowing pericardial effusion. An ophthalmology exam revealed no retinal pathology. The patient also underwent an infectious disease workup for HIV, hepatitis B, hepatitis C, syphilis, and tuberculosis with negative results.

**Table 2:** Rheumatologic workup RNP, ribonucleoproteins; DNA, deoxyribonucleic acid.

Autoimmune workup	Normal values	Results
Anti-nuclear antibodies	Negative	Positive
Anti-nuclear antibody titer	<1:40	1:1280
Anti-nuclear antibody pattern	None	Speckled
Anti-double-stranded DNA antibody	Negative	Positive
Anti-double-stranded DNA antibody titer	<1:10	1:5120
Anti-smith antibody	Negative	Positive
Anti-cardiolipin antibody	Negative	Negative
Lupus anticoagulant	Negative	Negative
Anti-beta-2-glycoprotein Ab, IgA IgG IgM	Negative	Negative
Serum C3 complement	90-180 mg/dL	27 mg/dL (low)
Serum C4 complement	15-45 mg/dL	2 mg/dL (low)
Rheumatoid factor-serum	Negative	Negative
Anti-RNP antibodies	Negative	Positive
Myeloperoxidase	Negative	Negative
Proteinase 3	Negative	Negative

The diagnosis of severe lupus flare was confirmed, and the patient was started on pulse dose steroids. A diagnostic cerebral angiogram revealed no evidence of cerebral vasculitis. No intervention was recommended from cardiothoracic surgery for pericardiocentesis. After a discussion with neurosurgery, the patient was started on a heparin drip for her bilateral pulmonary emboli and was later switched to apixaban 5 mg twice daily. A renal biopsy was deferred to the outpatient setting to evaluate for lupus nephritis. Guideline-directed medical therapy was initiated.

## Discussion

The symptoms and course of SLE vary greatly among patients; therefore, a comprehensive workup is essential to ensure timely diagnosis and tailor the treatment plan based on patients’ presentations. Our patient was recently diagnosed with SLE, presenting with weakness and dizziness six months ago. Magnetic resonance angiography of the circle of Willis showed cerebral vasculitis involving the anterior, posterior, and middle cerebral arteries bilaterally (Figure 1). Interestingly, she had no symptoms of neurological involvement during this current admission despite recent evidence of cerebral vasculitis.

The patient presented with isolated bilateral LE edema, and to our surprise, further workup uncovered the otherwise asymptomatic extensive involvement of multiple systems manifesting as bilateral pulmonary emboli, lupus pericarditis, and heart failure. The nephrotic or nephritic syndrome was suspected as the cause of the edema, as it has been reported in 45% to 65% of SLE patients [3], with proteinuria and hematuria present in 100% of those cases. A renal biopsy was therefore ordered to confirm the diagnosis; however, given the severity of the lupus flare, steroids were administered, necessitating deferment of the biopsy for the outpatient setting.

LE pitting edema in SLE is typically associated with renal or cardiac etiologies, both of which were evident in our patient. However, she presented with non-pitting edema instead. Differential diagnoses in this case primarily include lymphedema and pretibial myxedema [4]. In lymphedema, elevation of the extremity or diuretic therapy is inadequate and does not improve the swelling [5]. However, our patient reported improvement in her peripheral edema with leg elevation. Pretibial myxedema is associated with autoimmune thyroid disease or may be present in severe hypothyroidism; however, our patient did not present with any clinical symptoms of thyroid disease. Additionally, her TSH was within normal range. Of note, medications and DVT were ruled out as causes of her significant LE edema. Even though the findings of our case report are limited by the pending renal biopsy, nephrotic/nephritic syndrome does not explain non-pitting edema.

El-Shafie et al. reported a case of generalized edema as the initial and sole presenting symptom. In the absence of other reported causes of generalized edema associated with SLE, the authors concluded that generalized edema can be a rare cutaneous manifestation of SLE and can be the first and only manifestation of this disease [6]. Lu et al. suggested that immune complex deposition in the microvasculature and the change of vascular permeability may play a role in the pathogenesis of subcutaneous edema in an elderly patient with generalized edema and polyserositis in the absence of other clinical features of the disease [7]. Although the edema slightly improved after the administration of steroids and diuresis, the pathogenesis of non-pitting LE edema still remains unclear and warrants further investigation.

## Conclusions

SLE is a chronic autoimmune tissue disorder with heterogeneous presentations. Pitting edema of the lower limb and generalized subcutaneous edema with unclear etiology have been reported as possible rare manifestations of SLE; however, to our knowledge, isolated non-pitting LE edema in an otherwise asymptomatic patient with a severe underlying multisystem lupus flare has not. Patient education about the various manifestations of SLE, including such rare and non-classic symptoms, is essential to ensure the timely management of multisystem flares. In conclusion, the possibility of isolated LE edema as a symptom of multisystem SLE flare should be kept in mind, even in the absence of other clinical manifestations of the disease.
